# Establishment of Quantitative PCR Assays for Active Long Interspersed Nuclear Element-1 Subfamilies in Mice and Applications to the Analysis of Aging-Associated Retrotransposition

**DOI:** 10.3389/fgene.2020.519206

**Published:** 2020-09-16

**Authors:** Ryota Kuroki, Yui Murata, Satoshi Fuke, Yutaka Nakachi, Jun Nakashima, Gregory C. Kujoth, Tomas A. Prolla, Miki Bundo, Tadafumi Kato, Kazuya Iwamoto

**Affiliations:** ^1^ Department of Molecular Brain Science, Graduate School of Medical Sciences, Kumamoto University, Kumamoto, Japan; ^2^ Lab for Molecular Dynamics of Mental Disorders, RIKEN Center for Brain Science, Wako, Japan; ^3^ Department of Genetics and Medical Genetics, University of Wisconsin, Madison, WI, United States; ^4^ PRESTO, Japan Science and Technology Agency, Saitama, Japan

**Keywords:** retrotransposition, mitochondrial DNA, non-LTR, basal ganglia, somatic mosaicism, *POLG*, aging

## Abstract

The retrotransposon long interspersed nuclear element-1 (LINE-1) can autonomously increase its copy number within a host genome through the retrotransposition process. LINE-1 is active in the germline and in neural progenitor cells, and its somatic retrotransposition activity has a broad impact on neural development and susceptibility to neuropsychiatric disorders. The method to quantify the genomic copy number of LINE-1 would be important in unraveling the role of retrotransposition, especially in the brain. However, because of the species-specific evolution of LINE-1 sequences, methods for quantifying the copy number should be independently developed. Here, we developed a quantitative PCR (qPCR) assay to measure the copy number of active LINE-1 subfamilies in mice. Using the assay, we investigated aging-associated alterations of LINE-1 copy number in several brain regions in wild-type mice and *Polg^+/D257A^* mice as a model for accelerated aging. We found that aged *Polg^+/D257A^* mice showed higher levels of the type GfII LINE-1 in the basal ganglia than the wild-type mice did, highlighting the importance of assays that focus on an individual active LINE-1 subfamily.

## Introduction

Long interspersed nuclear element-1 (LINE-1) is a retrotransposon with a length of approximately 6 kb. It occupies approximately 17 and 19% of the human genome and mouse genome, respectively (Lander et al., 2001; Mouse Genome Sequencing Consortium et al., 2002). Full-length LINE-1 is composed of a 5' untranslated region (UTR), open reading frame (ORF) 1, ORF2, a 3'UTR, and a poly-A tail. ORF1 encodes an RNA-binding protein (Holmes et al., 1992; Hohjoh and Singer, 1997), and ORF2 encodes the protein with reverse transcriptase and endonuclease activity (Mathias et al., 1991; Feng et al., 1996). LINE-1 can increase its copy number within the host genome autonomously by a process called retrotransposition, which involves transcription of LINE-1, translation of ORFs, and translocating LINE-1 transcripts to the nucleus for their reverse transcription (Hohjoh and Singer, 1997; Cost et al., 2002). Retrotransposon activity is known to occur in germline cells and during early embryogenesis. When the newly transcribed copy of LINE-1 is inserted into genomic regions, it often affects genome stability and gene expression, resulting in a number of Mendelian diseases (Goodier and Kazazian, 2008; Cordaux and Batzer, 2009; Hancks and Kazazian, 2016). In addition to germline cells, recent findings suggest that LINE-1 is also active in neural precursor cells during early neurodevelopment and adult neurogenesis in the hippocampus, resulting in somatic mosaicism in brain cells (Erwin et al., 2014; Evrony et al., 2016; Faulkner and Billon, 2018; Saleh et al., 2019). Somatic LINE-1 retrotransposition in neurons is considered to be involved in the pathophysiology of neuropsychiatric disorders (Muotri et al., 2010; Coufal et al., 2011; Bundo et al., 2014; Iwamoto, 2019; Saleh et al., 2019).

In the other cell types and in most of the developmental stages, LINE-1 activity is strictly suppressed by multiple mechanisms, including genetic, epigenetic, posttranscriptional, and posttranslational regulation, depending on the type and evolutionary origin of LINE-1 (Goodier and Kazazian, 2008; Goodier, 2016). However, in addition to cancer (Rodic, 2018), accumulating evidence suggests that aging may be associated with increased LINE-1 activity (Saleh et al., 2019). The expression level and copy number of LINE-1 are increased with aging in liver and muscle tissue in mice (De Cecco et al., 2013; Min et al., 2019) and in senescent cells (De Cecco et al., 2019). An increase in LINE-1 copy number was also reported in the brains of adult rats compared to those of younger rats (Giorgi et al., 2018), and a similar increase was observed in mice with a deficiency in SIRT6, which is a regulator of longevity (Liao and Kennedy, 2016; Simon et al., 2019). Whether LINE-1 in nondividing mature neurons exhibits retrotransposition remains unclear, a study showed that engineered LINE-1 can retrotranspose in human neurons (Macia et al., 2017).

Estimation of the active LINE-1 copy number in human and model animals will provide important information for understanding the role of retrotransposition. For this purpose, a quantitative PCR (qPCR)-based estimation technique has been used, because it allows high-throughput measuring in a cost-effective manner. However, the structure and evolutionary characteristics of LINE-1 differ between humans and model animals, such as mice. In humans, only the most evolutionarily young LINE-1 subfamily, Hs, retains retrotransposition activity (Skowronski, et al., 1988), while at least three subfamilies (A, Gf, and Tf) retain activity in mice (Sookdeo et al., 2013). These three subfamilies are further classified into three A types (AI, AII, and AIII), two Gf types (GfI and GfII), and three Tf types (TfI, TfII, and TfIII). In addition, in mice, LINE-1 contains repeat sequences called monomers in the 5'UTR, which are not present in human LINE-1. Given that different active subfamilies in mice have different transcriptional activity and epigenetic profiles (DeBerardinis and Kazazian, 1999; Bulut-Karslioglu et al., 2014; Murata et al., 2017), detailed analysis of specific subfamilies is critically important.

Here, we developed a qPCR-based assay to quantify the copy number of active LINE-1 subfamilies in mice. Using this assay, we investigated aging-associated LINE-1 copy number change in *Polg^+/D257A^* mice, which we considered an animal model of chronic progressive external ophthalmoplegia (CPEO) that exhibits a premature aging characterized by accumulation of deleted mtDNA and motor dysfunction (Fuke et al., 2014). *POLG* is a nuclear-encoded mitochondrial DNA (mtDNA) polymerase, and its mutations are known to cause CPEO and associate with psychiatric disorders (Kasahara et al., 2017; Kato, 2019). Mice carrying a D257A knock-in mutation in *Polg* (*Polg^D257A/D257A^*) lost proofreading activity of mtDNA and showed drastic accelerated aging phenotypes, including weight loss, reduced subcutaneous fat, hair loss, curvature of the spine, osteoporosis, and a reduced life span (Trifunovic et al., 2004; Kujoth et al., 2005). Although the mice carrying the heterozygous *Polg^D257A^* (*Polg^+/D257A^*) were reportedly normal (Trifunovic et al., 2004; Kujoth et al., 2005), we previously found that they showed age-dependent increased accumulation of mtDNA deletions and behavioral alterations, including motor dysfunction (Fuke et al., 2014). In this study, we found a subfamily-specific increase in the LINE-1 copy number in the basal ganglia of aged *Polg^+/D257A^* mice, showing the importance of a specific assay focusing on an individual member of the LINE-1 subfamilies.

## Materials and Methods

### Primer Design

Consensus sequences of LINE-1 subfamilies in mice, including active subfamilies (Tf, A, and Gf) were retrieved from Repbase (Bao et al., 2015; Kojima, 2018). We designed both forward and reverse PCR primers with unique sequences for each active subfamily at their 3' ends. Primer sequences were searched for homologous consensus sequences using GENETYX ver.13 (GENETYX, Tokyo, Japan) to rule out the possibility of incorrect annealing. For the purpose of quality control, initial PCR was performed using rTaq DNA Polymerase (TOYOBO, Osaka, Japan) with a total of 5 ng of mouse genomic DNA as a template. PCR conditions were as follows: 1 min at 94°C followed by 40 cycles of 15 s at 95°C and 45 s at 65°C. Electrophoresis was performed on 2% agarose gel and visualized using GelGreen (COSMO BIO, Tokyo, Japan). Direct Sanger sequencing of PCR products was performed on all the candidate products after ExoSAP-IT Express PCR Cleanup Reagents (Thermo Fisher SCIENTIFIC, Waltham, Massachusetts, United States) were used to purify the DNA (Eurofins Genomics Inc., Tokyo, Japan).

### Quantitative PCR

qPCR was performed using THUNDERBIRD SYBR qPCR mix (TOYOBO) and a total of 500 pg of genomic DNA as a template; reactions were carried out on a Quantstudio® 5 Real-Time PCR System (Thermo Fisher SCIENTIFIC). All primer pairs were used at a 5 μM concentration. qPCR conditions were the same as those listed above. The melting curve analysis conditions were as follows: 15 s at 95°C, 15 s at 60°C, and 15 s at 95°C. LINE-1 copy number was adjusted using internal control, 5srRNA, used previously (Muotri et al., 2010; Bundo et al., 2014). Quantification was performed in triplicate per sample. Raw Ct data are available upon request.

### TA Cloning and Sequencing of Single Colonies

PCR products amplified with the GfII_ORF1 and GfI_5'UTR-ORF1 primer pairs (Table 1) were TA-cloned into a pCR4-TOPO vector using a TOPO TA cloning kit (Thermo Fisher SCIENTIFIC). We then transformed a DH5α strain with the vector samples and sequenced plasmids derived from single colonies.

**Table 1:** List of primer pairs used in this study.

Subfamily	Primer name		Sequence (5' -> 3')
Universal	m5UTR	F	TAAGAGAGCTTGCCAGCAGAGA
		R	GCAGACCTGGGAGACAGATTCT
	mORF1	F	TGGAAGAGAGAATCTCAGGTGC
		R	TTGTGCCGATGTTCTCTATGG
	mORF2	F	CTGGCGAGGATGTGGAGAA
		R	CCTGCAATCCCACCAACAAT
AI, AII	A_ORF2_1	F	CACTTTAGTAAAGCTCAAAGCAT
		R	ATGTTCTGTAGATATCTGTCAGG
AI, AII, AIII	A_ORF1	F	GACCAAACCTACGGATAATAGGAATT
		R	GATCATGGGCATCTCTTTTTTTAT
	A_ORF2_2	F	TTGGCGTGACTCTAACTAAGGAG
		R	CCTAGGTTTTTTGTTATTCCAGACA
GfI	GfI_5'UTR-ORF1	F	AGAGAGCTTGTCTCCCACGC
		R	CATGAGATATGCTTTTAAATCCAGGTCTAC
GfII	GfII_ORF1	F	AACCCAAAGTGAGGCAACAG
		R	CATCCACTCCTA TTATCCGTAGGTTC
TfII	TfII_3'UTR	F	GGGATCCACCCCATAATCAGCTTCCAAAT
		R	TCCCCTGTACCGGGGCACAC
Internal control	m5srRNA	F	ACGGCCATACCACCCTGAA
		R	GGTCTCCCATCCAAGTACTAACCA

Universal and internal control pairs were previously reported (Muotri et al., 2010; Bundo et al., 2014).

### Dendritic Tree

Dendritic trees were drawn using the mouse LINE-1 consensus sequences, using NJplot (Perrière and Gouy, 1996) based on maximum likelihood phylogenetic tree by PsyML (Dereeper et al., 2008). The tree of PCR amplicons (*N* = 65 for GFII_ORF1 and *N* = 49 for GfI_5'UTR-ORF1) was derived from TA-cloning, using GENETYX ver.13 based on neighbor joining method and a Kimura 2-parameter model. Three consensus sequences (L1MM_F, L1VL1_5, and L1VL2_5) were removed from these analyses because they did not have a corresponding sequence to the target region.

### Animal Model

*Polg^D257A^* mice were described previously (Kujoth et al., 2005; Fuke et al., 2014). In brief, *Polg^+/D257A^* mice (Kujoth et al., 2005) were backcrossed with C57BL/6JJcl mice for more than six generations (Fuke et al., 2014). Five brain regions, frontal lobe, hippocampus, posterior cortex, basal ganglia, and cerebellum were dissected, and genomic DNAs were extracted from them as described (Fuke et al., 2014).

### Statistical Analysis

Welch’s *t*-test was employed for comparison between two groups. *p* < 0.05 was considered significant. We considered a robust change to occur only if changes in both tissues were supported by statistical significance (Welch’s *t*-test, *p* < 0.05 in both tissues).

## Results

### Establishment of qPCR Assays for Individual Active LINE-1 Subfamilies in Mice

**Figure 1:**
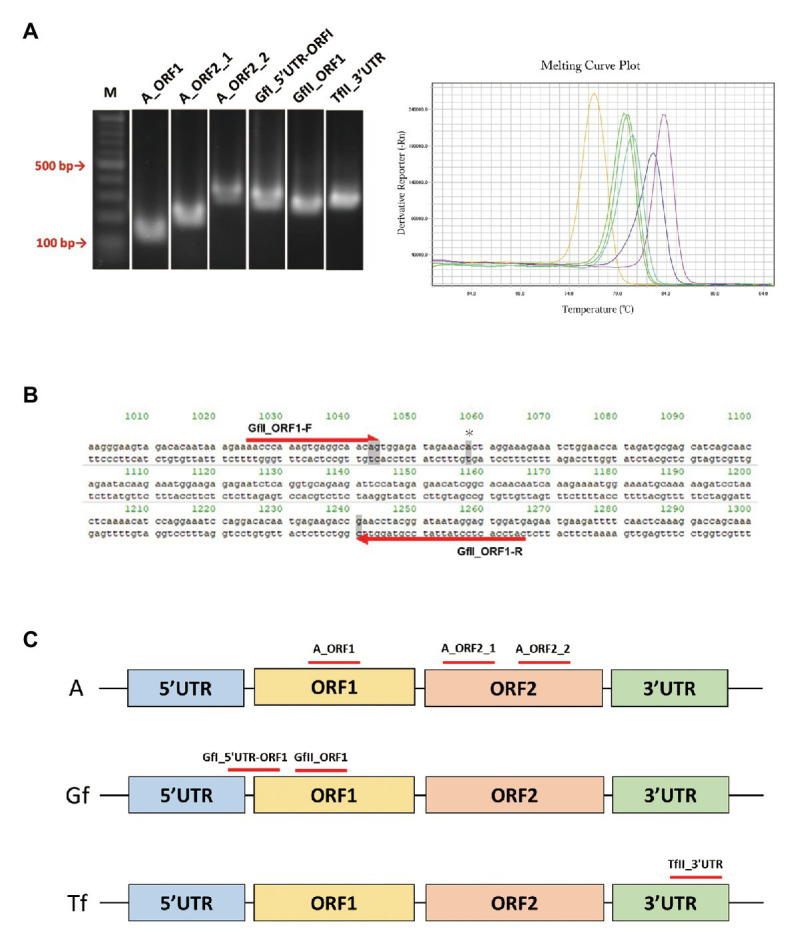
PCR primer pairs specific for individual active long interspersed nuclear element-1 (LINE-1) subfamilies. **(A)** Agarose gel electrophoresis of PCR products, *left*. Melting curve analysis of PCR products in the quantitative PCR (qPCR) context, *right*. M, molecular size markers. **(B)** Sequences of the GfII_ORF1 primer pair. Primer sequences aligned with the consensus sequence of GfII are shown. The unique sequence in GfII is highlighted in gray. ^*^Indicates the unique sequence used for sequencing analysis of PCR products. **(C)** Location of the validated primer pairs. Monomer sequencers are omitted from the illustrations.

We retrieved a total of 34 mouse LINE-1 consensus sequences from Repbase, which is a database of repetitive DNA elements (Bao et al., 2015; Kojima, 2018). Based on the consensus sequences, primer pairs that can specifically amplify the active LINE-1 subfamily (A, Gf, and Tf) were designed. Because each active subfamily was further divided into subtypes, i.e., A for AI, AII, and AIII, Gf for GfI and GfII, and Tf for TfI, TfII, and TfIII (Supplementary Figure S1), we first tried to design primer pairs for each type and then designed primers to include several types within the same subfamily. After excluding the primer pairs that may incorrectly anneal to other LINE-1 locations, we designed a total of 28 primer pairs, including four pairs for AI, AII, and AIII, one pair for AI and AII, four pairs for GfI, eight pairs for GfII, two pairs for TfI, one pair for TfII, three pairs for TfIII, and five pairs for TfI and TfII. We then determined whether the designed primer pair produced a single amplicon by agarose gel electrophoresis (Figure 1A), followed by direct Sanger sequencing. Representative data of Gf_II ORF1 were shown in Supplementary Figure S2. Primer pairs were then tested using a melting curve analysis in a qPCR context (Figure 1A). Amplicons from the primer pairs, GfII_ORF1, and GfI_5'UTR-ORF1 (Figures 1B,C and Table 1) were further analyzed by TA-cloning. In GfII_ORF1, sequences obtained from 65 individual bacterial colonies revealed that 81.5% (53/65) of amplicons showed high similarity with the GfII consensus sequence. Other amplicons (12/65) were also considered to be GfII variants because all of them contained unique GfII-specific sequences (Supplementary Figure S2). Similarly, in GfI_5'UTR-ORF1, sequences obtained from 49 individual bacterial colonies revealed that 79.6% (39/49) of amplicons showed high similarity with the GfI consensus sequence. All other amplicons (10/49) were also considered to be GfI variants because they contained unique GfI-specific sequences (data not shown). Finally, we obtained a total of six primer pairs that were highly specific for the target active LINE-1 subfamilies. They included two pairs for all three active type A (I, II, and III), one pair for AI and AII, and one pair each for GfI, GfII, and TfII (Figure 1C and Table 1).

### Subfamily-Specific LINE-1 Copy Number Analysis of Various Brain Regions of *Polg^+/D257A^* Mice

**Figure 2:**
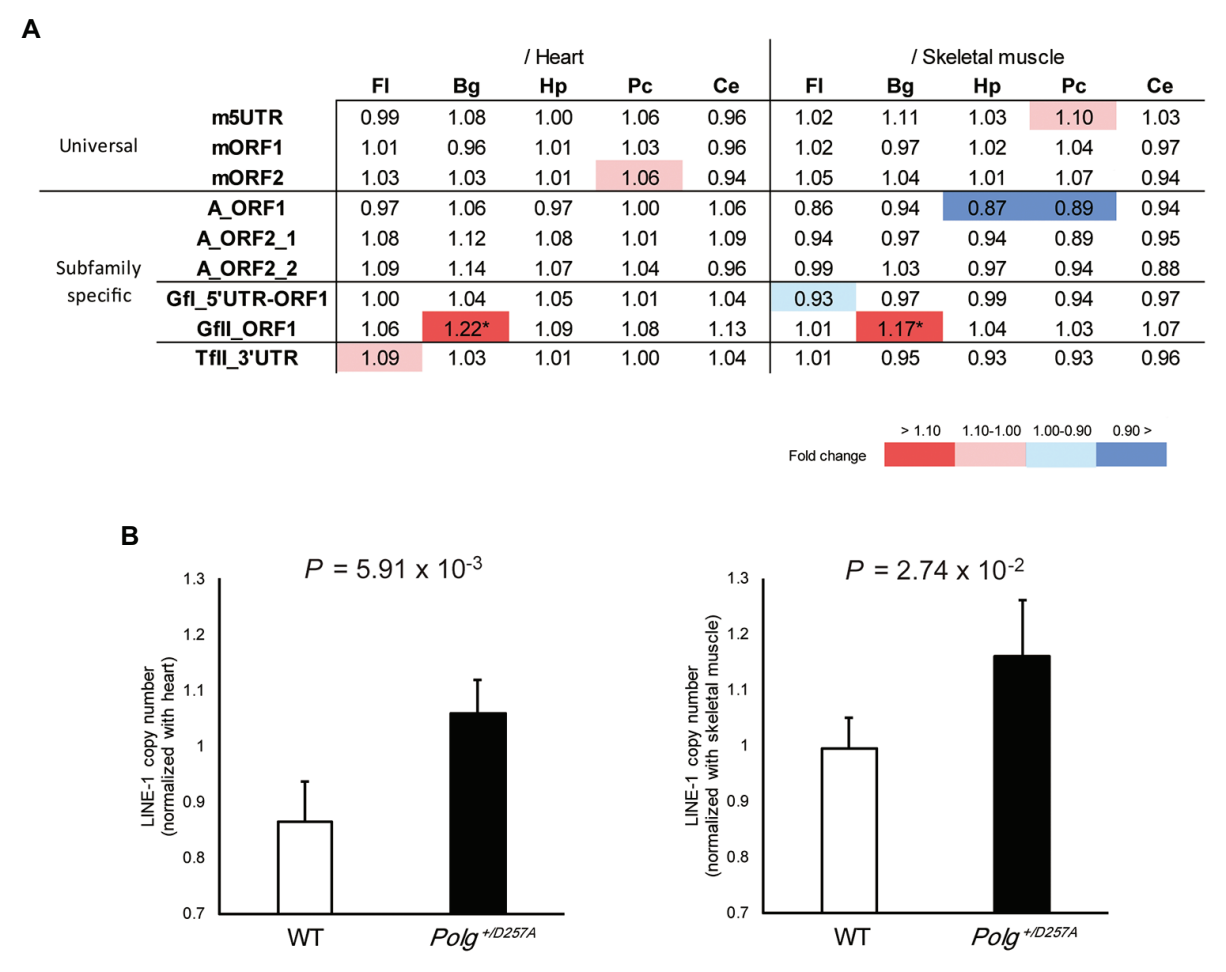
LINE-1 copy number detected in 84-week-old *Polg^+/D257A^* mouse brain. **(A)** Changes in LINE-1 content in *Polg^+/D257A^* mice (*n* = 4) and wild-type mice (*n* = 4). The fold change relative to wild-type mice is shown in each reference tissue. Color indicates the extent of fold change with a nominal significant difference (Welch’s *t*-test, *p* < 0.05). ^*^Indicates a robust change defined as significant in both references (Welch’s *t*-test, *p* < 0.05 in both tissues). Fl, frontal lobe; Hp, hippocampus; Pc, posterior cortex; Bg, basal ganglia; Ce, cerebellum. **(B)** LINE-1 copy number in the basal ganglia measured using the primer pair GfII_ORF1. The copy number in the brain was normalized to the number in the heart (left panel) or skeletal muscle (right panel). Data are represented as the mean ± standard deviation. WT, wild-type mice. All the comparisons were listed in Supplementary Figure S3.

We measured LINE-1 copy number in various brain regions (basal ganglia, cerebellum, frontal lobes, hippocampus, and posterior cortex) from the aged *Polg^+/D257A^* mice; these mice were shown to exhibit accumulation of mtDNA deletions during aging (Fuke et al., 2014). We examined the LINE-1 copy number in aged mice (84 weeks old) with conventional primer pairs that do not target specific LINE-1 subfamilies (Muotri et al., 2010; Bundo et al., 2014) and those we developed in this study (Table 1). Due to the multiple statistical testing methods and the limited number of samples, we used two different tissues, heart and skeletal muscles, as references. We considered a robust change to occur only if changes in both tissues were supported by statistical significance (Welch’s *t*-test, *p* < 0.05 in both tissues). We found that the conventional primer pairs did not detect copy number changes in the tested brain regions from *Polg^+/D257A^* mice (Figure 2A). However, among the developed primer pairs, we found consistently higher GfII LINE-1 copy numbers in the basal ganglia in *Polg^+/D257A^* mice than in wild-type mice (Figures 2A,B). All the comparisons were listed in Supplementary Figure S3.

## Discussion

Here, we developed subfamily-specific LINE-1 copy number assays in mice and investigated age-related changes in LINE-1 copy number in the brains of *Polg^+/D257A^* mice. We found that aged *Polg^+/D257A^* mice showed an increase of GfII in the basal ganglia over what was seen in wild-type mice, highlighting the importance of specific assays focusing on individual active LINE-1 subfamilies.

We successfully generated a total of six primer pairs that were highly specific to target subfamilies. Copy number and expression analyses specifically targeting active LINE-1 subfamilies in mice were previously reported (Jachowicz et al., 2017; Bedrosian et al., 2018). However, the primers in those studies were designed to amplify conserved regions among three active subfamilies (Bedrosian et al., 2018) or monomer regions (Jachowicz et al., 2017), which were located in the upstream region of the 5'-UTR of LINE-1; thus, those regions were not suitable for measuring somatic retrotransposition because the reverse transcription process usually stops prematurely.

In quantifying repetitive sequences such as LINE-1 by qPCR, the sequence specificity has been the critical confounding factor (Evrony et al., 2016). Based on previous reports, the total target LINE-1 copy numbers are estimated to be 3,466 for type A, 615 for GfI, 368 for GfII, and 1,282 for TfII in the full-length context (Sookdeo et al., 2013). Moreover, subfamily specificity of LINE-1 is important from a functional point of view. Transcriptional level of LINE-1 is proportional to the number of monomers in the 5'UTR (DeBerardinis and Kazazian, 1999), which are different in each subfamily. Epigenetic status, including DNA methylation and histone markers, is distinct in each LINE-1 subfamily (Bulut-Karslioglu et al., 2014; Murata et al., 2017). The subfamily specificity found in this study further supports the distinct regulation of LINE-1 retrotransposition activity in mouse brain cells.

It is noteworthy that in the protocol described here, we used 500 pg of genomic DNA as a template for qPCR. However, we confirmed that stable quantification data can be obtained from 100 pg of genomic DNA. Thus, the analysis is possible in more specific anatomical brain regions or in smaller cell populations. We also confirmed that the described primer pairs can be used for measuring subfamily-specific expression levels (Murata et al., unpublished data).

Several limitations should be kept in mind in applying the primers, however. First, because we put the highest priority in selecting primer sequences with high specificity for a target subfamily, some types within a subfamily were not assessed, or they were measured together. In the A subfamily, we obtained primer pairs common to AI, AII, and AIII, and a primer pair common to AI and AII. In the Gf subfamily, the primer pairs specific for GfI or GfII were independently established. In the Tf subfamily, the established primer pair measured TfII but not TfI or TfIII. Therefore, the results should be interpreted depending on the covered types. Second, the locations of the amplified regions differed among the primer pairs. Considering that the reverse transcription process is immaturely ended in general, the primer pairs targeted for the 3' end of LINE-1 would have more sensitivity for detecting retrotransposition events, whereas those targeting the upstream region of LINE-1 could examine more functional retrotransposition. Therefore, the sensitivity of the measured data will be different based on the target location of the primers. Third, although SYBR-based qPCR has been widely used for LINE-1 copy number assay (Muotri et al., 2010; Bundo et al., 2014), other quantification approaches such as the Taqman-probes, the peptide nucleic acid-probes, and the droplet digital PCR technique (Newkirk et al., 2020) will improve the sensitivity and the specificity of LINE-1 copy number assay.

Increased activity of LINE-1 in aging and senescent cells has been reported (De Cecco et al., 2013, 2019; Liao and Kennedy, 2016; Giorgi et al., 2018; Min et al., 2019; Saleh et al., 2019; Simon et al., 2019). Our data showing an increased GfII copy number in the basal ganglia of aged *Polg^+/D257A^* mice seemed to be in accordance with these previous reports. *Polg^D257A/D257A^* mice showed a severe phenotype of premature aging, resulting in premature death starting at 40 weeks (Trifunovic et al., 2004; Kujoth et al., 2005), so we analyzed the heterozygous mutant in this study. Although the phenotypes of *Polg^+/D257A^* mice were reportedly normal, we previously observed the presence of mild motor dysfunction at 34 weeks and the accumulation of deleted mtDNAs from 48 weeks in the basal ganglia without reducing the life span.

Among the various brain regions we analyzed, we detected robust LINE-1 copy number change in the basal ganglia. Basal ganglia have a relatively higher number of mtDNAs compared to other brain regions (Fuke et al., 2014); thus, it may be a susceptible brain region to aging-related LINE-1 copy number change. Each active LINE-1 subfamily harbors unique structures of monomers, tandem repeats in the promoter regions and different epigenetic status in brain (Murata et al., 2017). These suggested that they have different expression pattern and distinctive roles. Therefore, increased GfII in basal ganglia during aging suggests that there might be GfII-specific regulators in basal ganglia, whose expressions were altered during aging.

We detected 1.1-fold change in GfII by qPCR. The standard curve analysis indicated that Ct values showed a linear relationship around this magnitude of change (data not shown). By roughly estimation, this change corresponds to increase of about 37 copies of GfII per cell. The copy number change of this magnitude has been often reported by qPCR analyses of LINE-1 (Coufal et al., 2009, for example). However, genome analyses of single neurons reported much smaller extent of changes that cannot be theoretically detected by qPCR (Evrony et al., 2012, 2016; Sanchez-Luque et al., 2019). Other approaches such as deep sequencing analysis will help to interpret the possible discrepancy.

Accumulation of deleted mtDNA has been observed in heart and skeletal muscles (Fuke et al., 2014). Because we used these tissues as references in this study, our copy number estimation in brain may be confounded, if these tissues showed altered LINE-1 activities. However, we did not detect LINE-1 copy number change in heart normalized by skeletal muscle (and vice versa) between *Polg^+/D257A^* and wild-type mice (Supplementary Figure S4).

In senescent cells, an increased LINE-1 copy number is concomitant with increased expression of LINE-1, which is driven by increased expression of the activator FOXA1, decreased expression of the repressor RB1, and LINE-1 demethylation (De Cecco et al., 2019; Min et al., 2019). In addition, LINE-1 copy number in the cytosol is further increased by decreased TREX1 3' exonuclease (Thomas et al., 2017; De Cecco et al., 2019). A similar scenario in the brain of this aging mice model might be applicable. Future experiments will include examination of expression levels of the relevant genes, epigenetic status of LINE-1, and LINE-1 copy number in cytosolic DNA for better understanding of the role of retrotransposition and aging.

## Data Availability Statement

Data used in preparing this article can be available upon request.

## Ethics Statement

The animal study was reviewed and approved by the Animal Experiment Committee of RIKEN (Wako, Saitama, Japan) and Kumamoto University (Kumamoto City, Kumamoto, Japan).

## Author Contributions

RK and YM equally contributed to the work. RK, YM, YN, and JN performed the experiments and data analyses. SF, GK, and TP provided the materials. TK, MB, and KI supervised the study. RK, YM, and KI wrote the manuscript. All authors contributed to the article and approved the submitted version.

### Conflict of Interest

The authors declare that the research was conducted in the absence of any commercial or financial relationships that could be construed as a potential conflict of interest.
