# Supplementary material for: Establishment of Quantitative PCR Assays for Active Long Interspersed Nuclear Element-1 Subfamilies in Mice and Applications to the Analysis of Aging-Associated Retrotransposition
Source: Front Genet. 2020 Sep 16;11:519206. doi: 10.3389/fgene.2020.519206 (PMC7525186; doi:10.3389/fgene.2020.519206)
Supplement: Supplementary file 1 [file Data_Sheet_1.pdf]

Supplementary Material

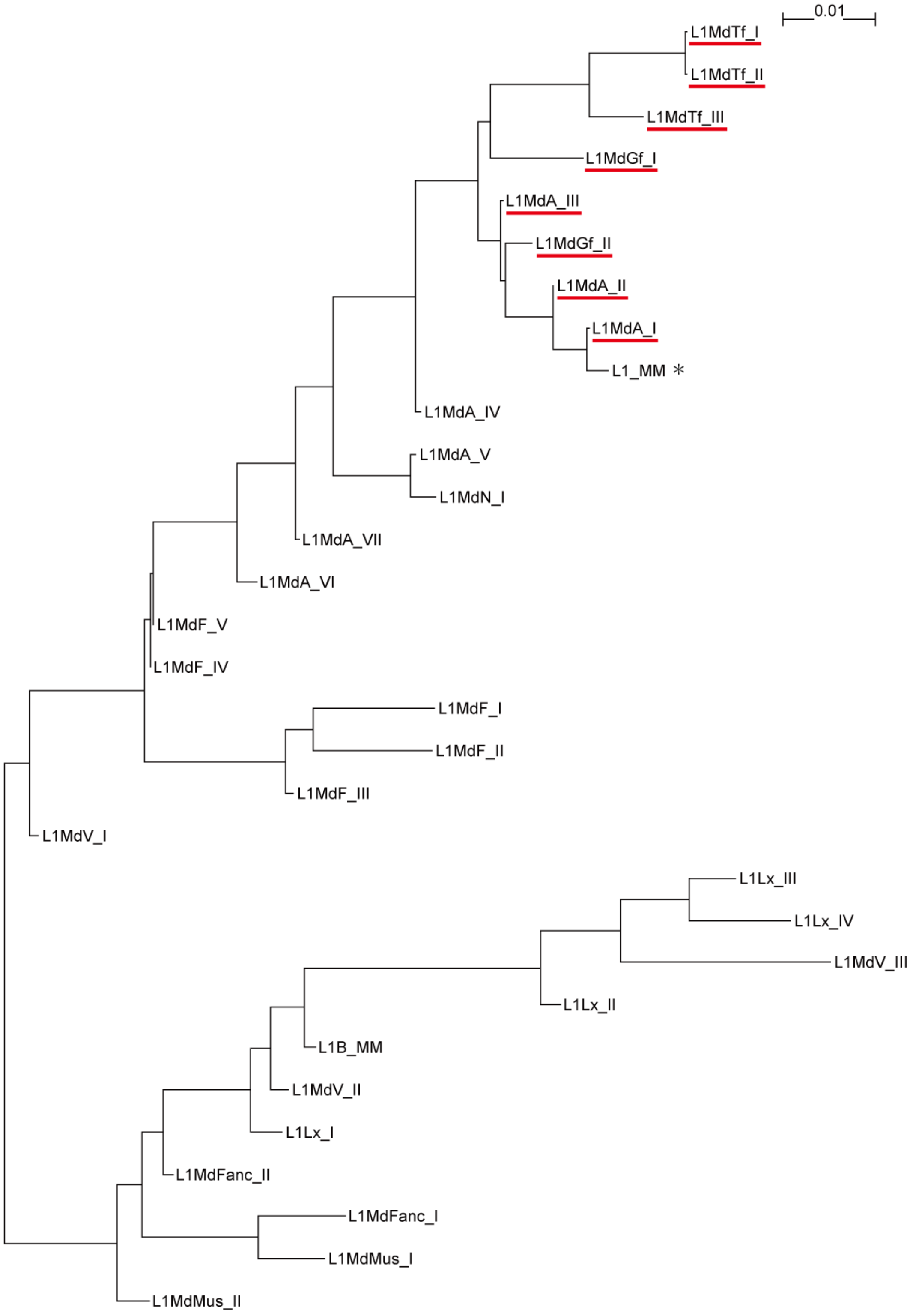

Figure S1. Dendritic tree of mouse L1 subfamily based on ORF2. Red underlines indicate subfamilies retaining retrotransposition activity. * is an ancestor LINE-1 sequence predicted from the confirmed LINE-1 (Shehee et al., 1987).

(A)

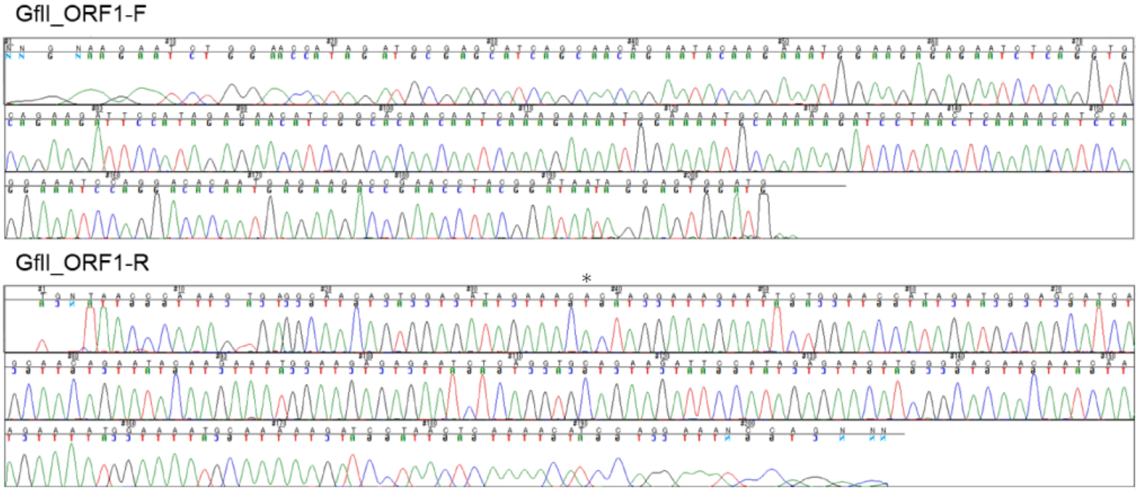
GfII_ORF1-F
GfII_ORF1-R
*

(B)

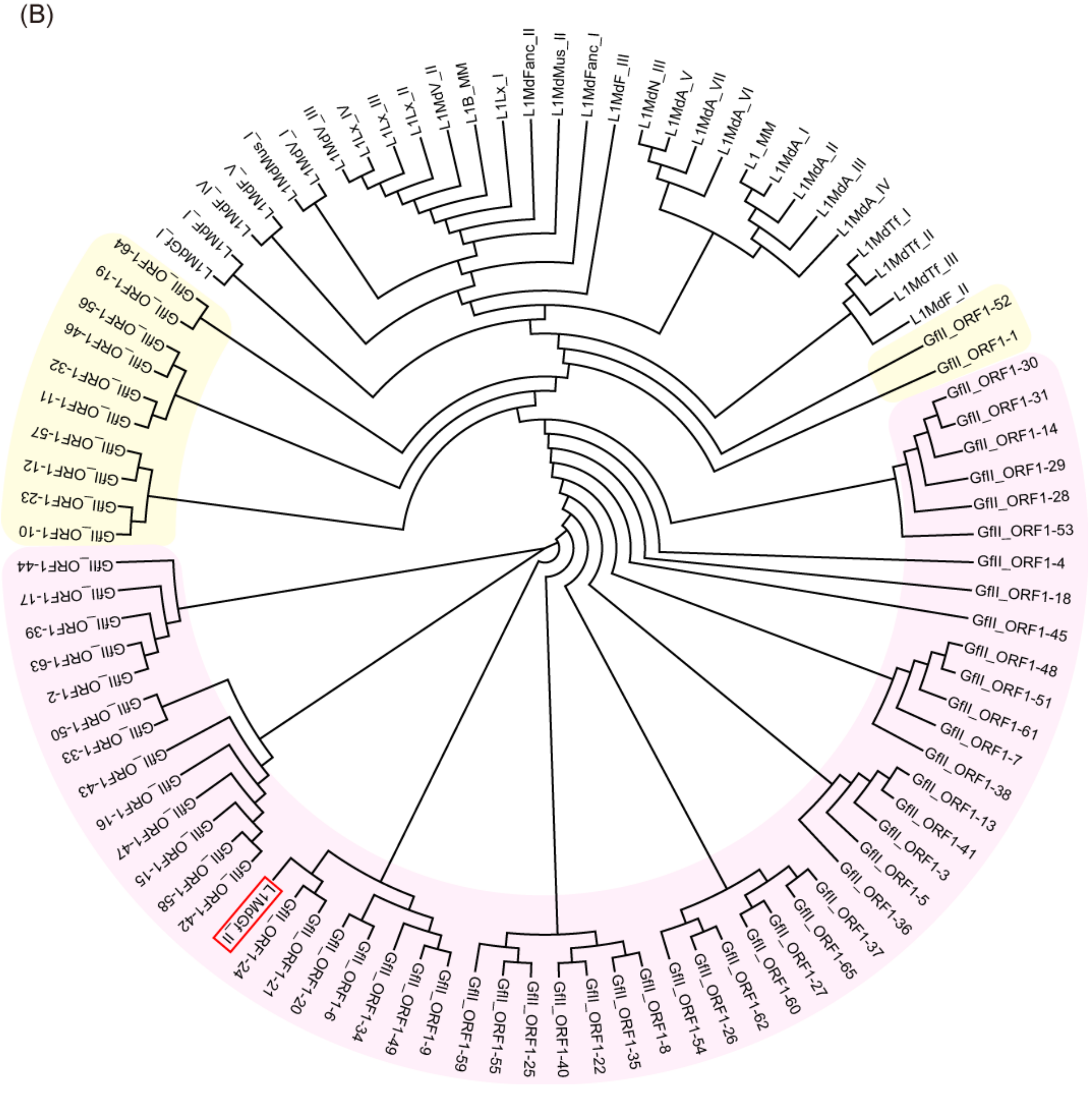
L1MdFanc_II
L1MdMus_II
L1MdFanc_I
L1MdF_III
L1MdN_III
L1MdA_V
L1MdA_VII
L1MdA_VI
L1_MM
L1MdA_I
L1MdA_II
L1MdA_III
L1MdA_IV
L1MdTf_I
L1MdTf_II
L1MdTf_III
L1MdF_II
GfII_ORF1-52
GfII_ORF1-1
GfII_ORF1-30
GfII_ORF1-31
GfII_ORF1-14
GfII_ORF1-29
GfII_ORF1-28
GfII_ORF1-53
GfII_ORF1-4
GfII_ORF1-18
GfII_ORF1-45
GfII_ORF1-48
GfII_ORF1-51
GfII_ORF1-61
GfII_ORF1-7
GfII_ORF1-38
GfII_ORF1-13
GfII_ORF1-41
GfII_ORF1-3
GfII_ORF1-5
GfII_ORF1-36
GfII_ORF1-37
GfII_ORF1-65
GfII_ORF1-27
GfII_ORF1-60
GfII_ORF1-62
GfII_ORF1-26
GfII_ORF1-54
GfII_ORF1-8
GfII_ORF1-35
GfII_ORF1-22
GfII_ORF1-40
GfII_ORF1-25
GfII_ORF1-55
GfII_ORF1-59
GfII_ORF1-9
GfII_ORF1-49
GfII_ORF1-34
GfII_ORF1-6
GfII_ORF1-20
GfII_ORF1-21
GfII_ORF1-24
L1MdGf_II
GfII_ORF1-42
GfII_ORF1-58
GfII_ORF1-15
GfII_ORF1-47
GfII_ORF1-16
GfII_ORF1-43
GfII_ORF1-33
GfII_ORF1-50
GfII_ORF1-2
GfII_ORF1-63
GfII_ORF1-39
GfII_ORF1-17
GfII_ORF1-44
GfII_ORF1-10
GfII_ORF1-23
GfII_ORF1-12
GfII_ORF1-57
GfII_ORF1-11
GfII_ORF1-32
GfII_ORF1-46
GfII_ORF1-56
GfII_ORF1-19
GfII_ORF1-64
L1MdGf_I
L1MdF_I
L1MdF_IV
L1MdF_V
L1MdMus_I
L1MdV_I
L1MdV_III
L1Lx_IV
L1Lx_III
L1Lx_II
L1MdV_II
L1B_MM
L1Lx_I

Figure S2. Sequencing analysis of the PCR products amplified with the GfII_ORF1 primer pair. (A) Results of direct sequencing of PCR products. * is the sequence specific for GfII. (B) Dendrogram of 65 sequences derived from single colonies and 31 mouse LINE-1 consensus sequences. Amplicons of the GfII ORF1 are highlighted in pink. Clones that contained GfII-specific sequences are highlighted in yellow. The consensus sequence for GfII is shown in the red rectangle.

(A) m5UTR

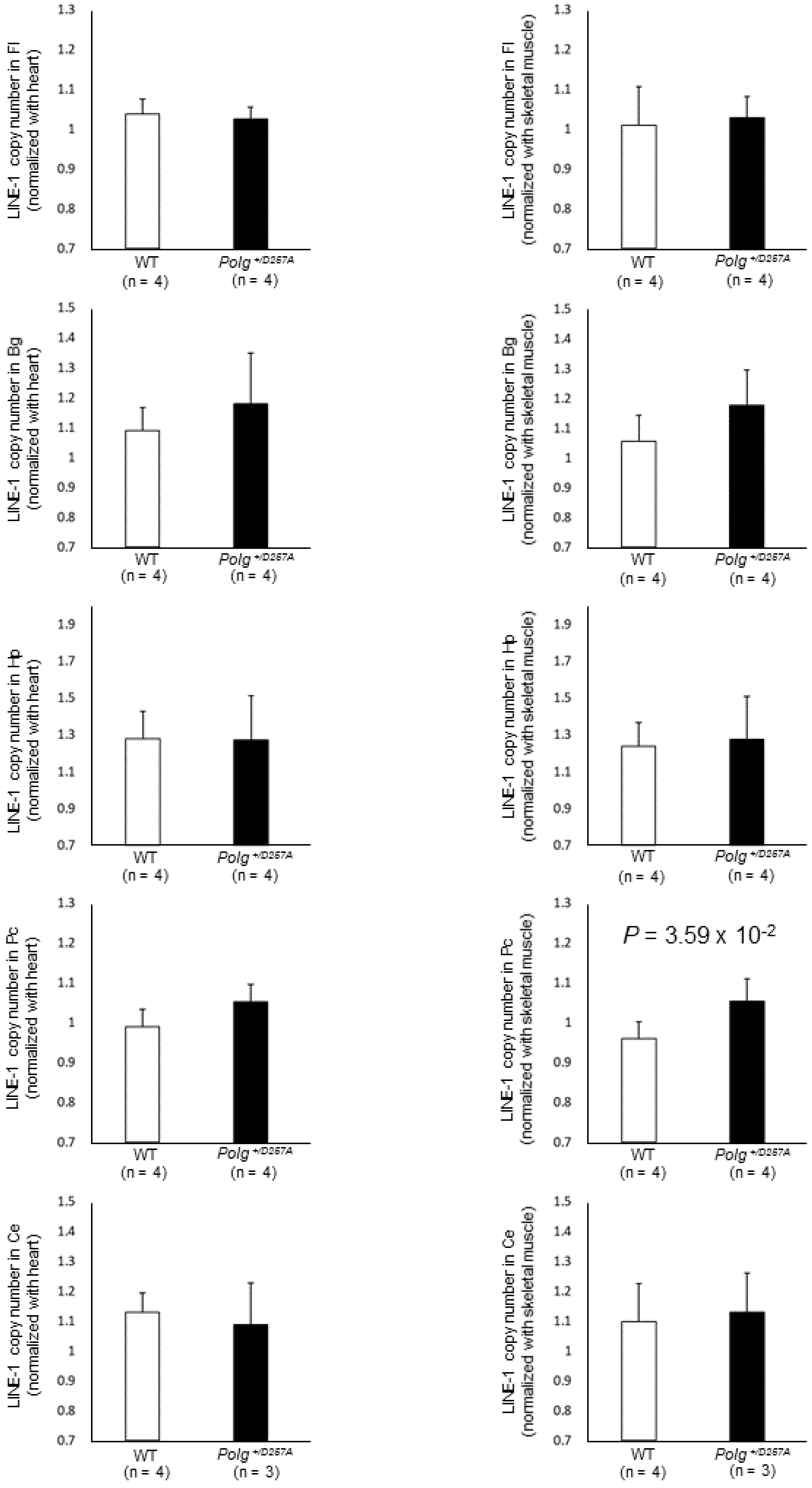
LINE-1 copy number in Fl (normalized with heart)
LINE-1 copy number in Fl (normalized with skeletal muscle)
LINE-1 copy number in Bg (normalized with heart)
LINE-1 copy number in Bg (normalized with skeletal muscle)
LINE-1 copy number in Hp (normalized with heart)
LINE-1 copy number in Hp (normalized with skeletal muscle)
LINE-1 copy number in Pc (normalized with heart)
LINE-1 copy number in Pc (normalized with skeletal muscle)
$P = 3.59 \times 10^{-2}$
LINE-1 copy number in Ce (normalized with heart)
LINE-1 copy number in Ce (normalized with skeletal muscle)
WT (n = 4)
Polg +/D257A (n = 4)
Polg +/D257A (n = 3)

(B) mORF1

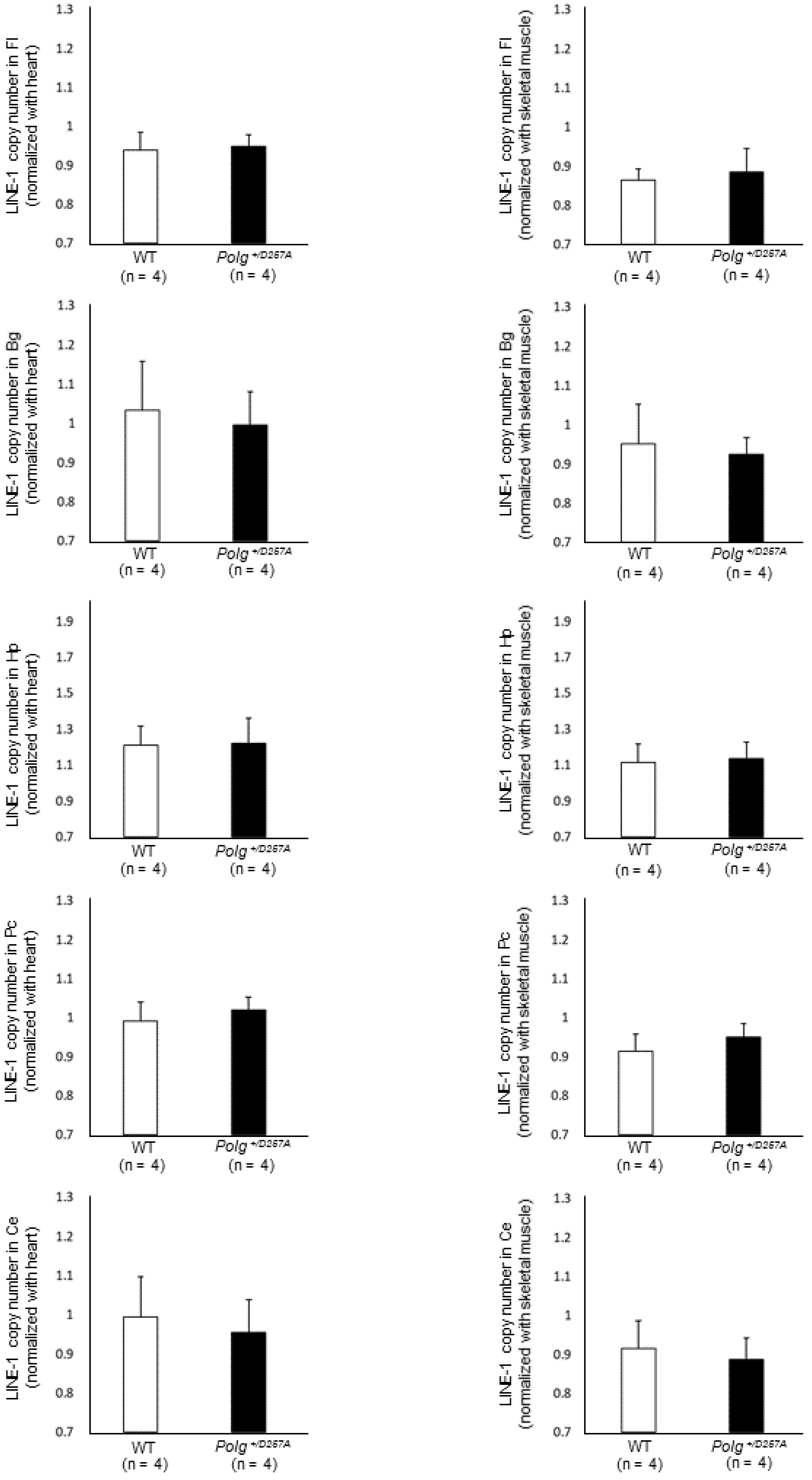

LINE-1 copy number in Fl (normalized with heart)
LINE-1 copy number in Fl (normalized with skeletal muscle)
LINE-1 copy number in Bg (normalized with heart)
LINE-1 copy number in Bg (normalized with skeletal muscle)
LINE-1 copy number in Hp (normalized with heart)
LINE-1 copy number in Hp (normalized with skeletal muscle)
LINE-1 copy number in Pc (normalized with heart)
LINE-1 copy number in Pc (normalized with skeletal muscle)
LINE-1 copy number in Ce (normalized with heart)
LINE-1 copy number in Ce (normalized with skeletal muscle)
1.3
1.2
1.1
1
0.9
0.8
0.7
1.9
1.7
1.5
1.3
1.1
0.9
0.7
WT (n = 4)
Polg +/D257A (n = 4)

(C) mORF2

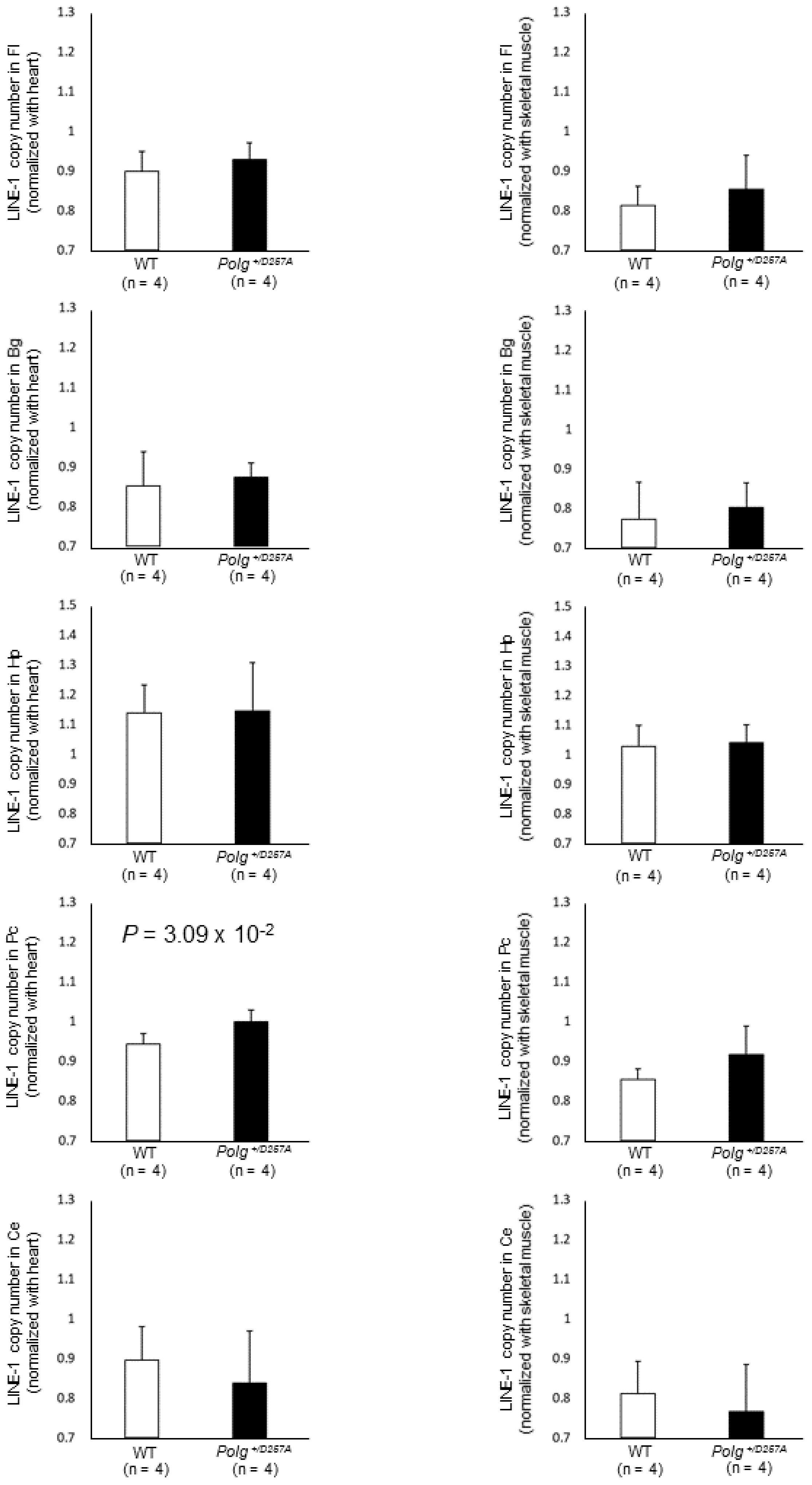

LINE-1 copy number in Fl (normalized with heart)
LINE-1 copy number in Fl (normalized with skeletal muscle)
LINE-1 copy number in Bg (normalized with heart)
LINE-1 copy number in Bg (normalized with skeletal muscle)
LINE-1 copy number in Hp (normalized with heart)
LINE-1 copy number in Hp (normalized with skeletal muscle)
LINE-1 copy number in Pc (normalized with heart)
P = 3.09 x 10^{-2}
LINE-1 copy number in Pc (normalized with skeletal muscle)
LINE-1 copy number in Ce (normalized with heart)
LINE-1 copy number in Ce (normalized with skeletal muscle)
WT (n = 4)
Polg $^{+/D257A}$ (n = 4)

(D) A_ORF1

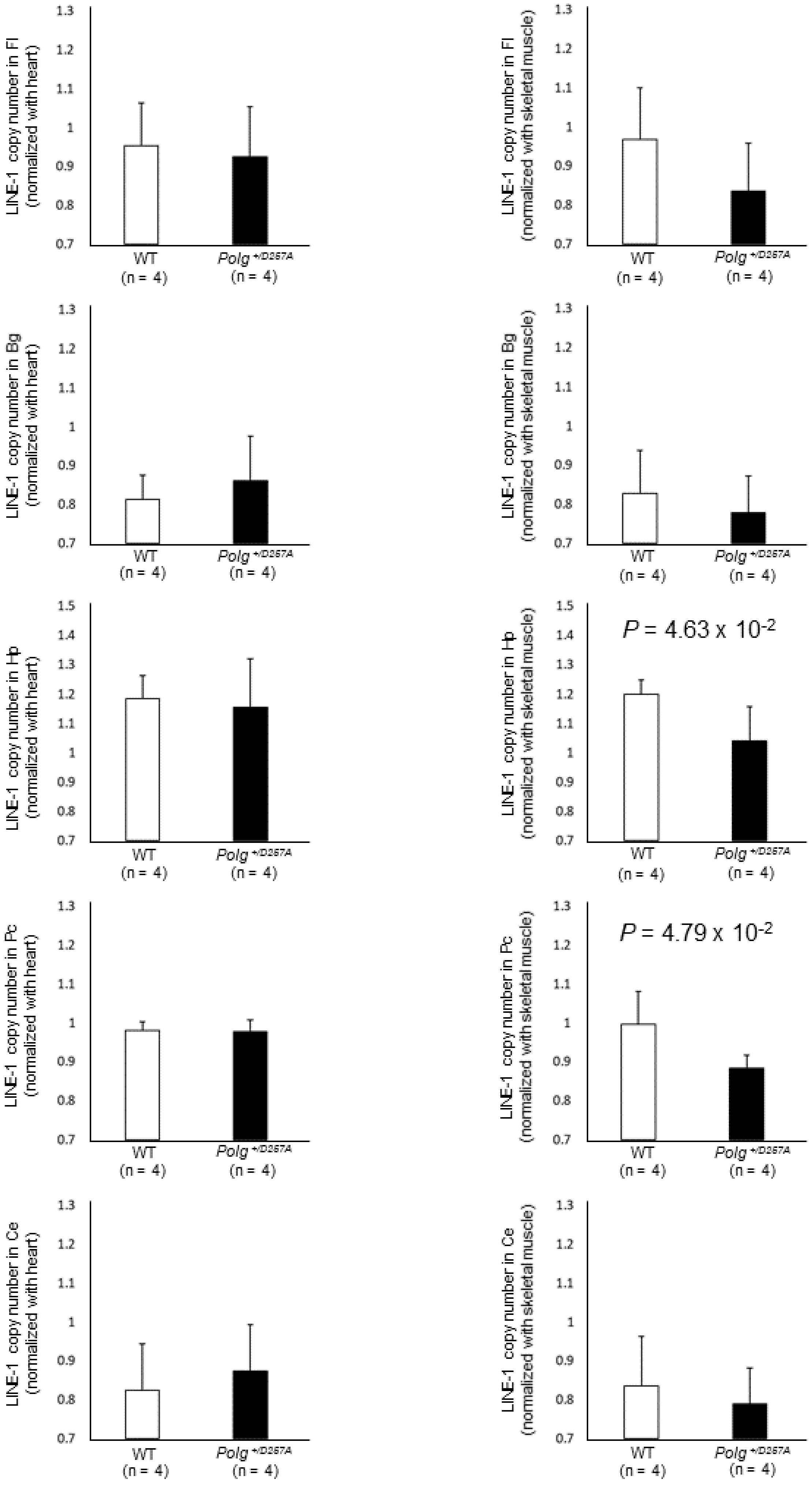
LINE-1 copy number in Fl (normalized with heart)
LINE-1 copy number in Fl (normalized with skeletal muscle)
LINE-1 copy number in Bg (normalized with heart)
LINE-1 copy number in Bg (normalized with skeletal muscle)
LINE-1 copy number in Hp (normalized with heart)
LINE-1 copy number in Hp (normalized with skeletal muscle)
P = 4.63 x 10-2
LINE-1 copy number in Pc (normalized with heart)
LINE-1 copy number in Pc (normalized with skeletal muscle)
P = 4.79 x 10-2
LINE-1 copy number in Ce (normalized with heart)
LINE-1 copy number in Ce (normalized with skeletal muscle)
WT
(n = 4)
Polg +/D257A
(n = 4)

(E) A_ORF2_1

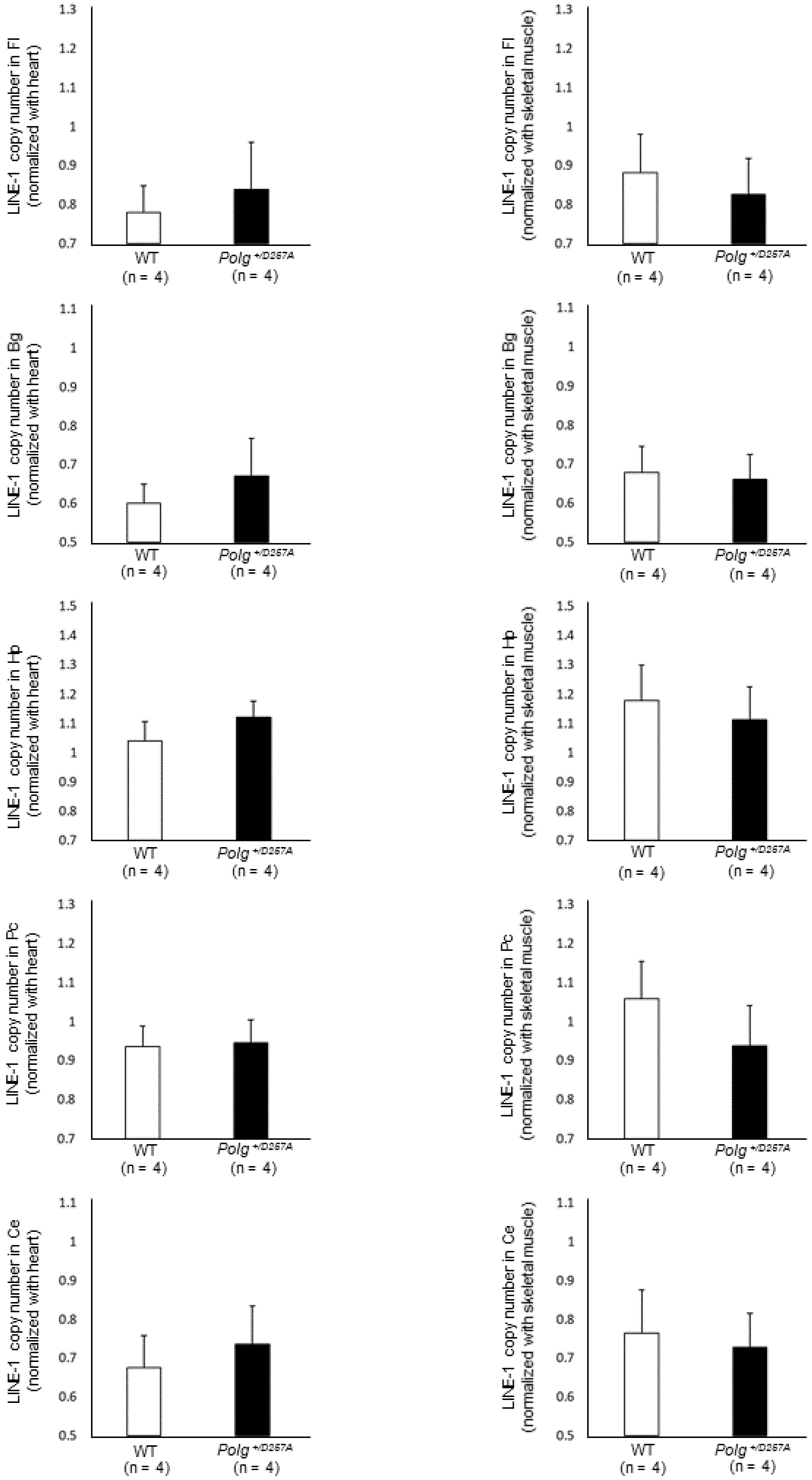

LINE-1 copy number in Fl
(normalized with heart)
LINE-1 copy number in Fl
(normalized with skeletal muscle)
LINE-1 copy number in Bg
(normalized with heart)
LINE-1 copy number in Bg
(normalized with skeletal muscle)
LINE-1 copy number in Hp
(normalized with heart)
LINE-1 copy number in Hp
(normalized with skeletal muscle)
LINE-1 copy number in Pc
(normalized with heart)
LINE-1 copy number in Pc
(normalized with skeletal muscle)
LINE-1 copy number in Ce
(normalized with heart)
LINE-1 copy number in Ce
(normalized with skeletal muscle)
WT
(n = 4)
Polg +/D257A
(n = 4)

(F) A_ORF2_2

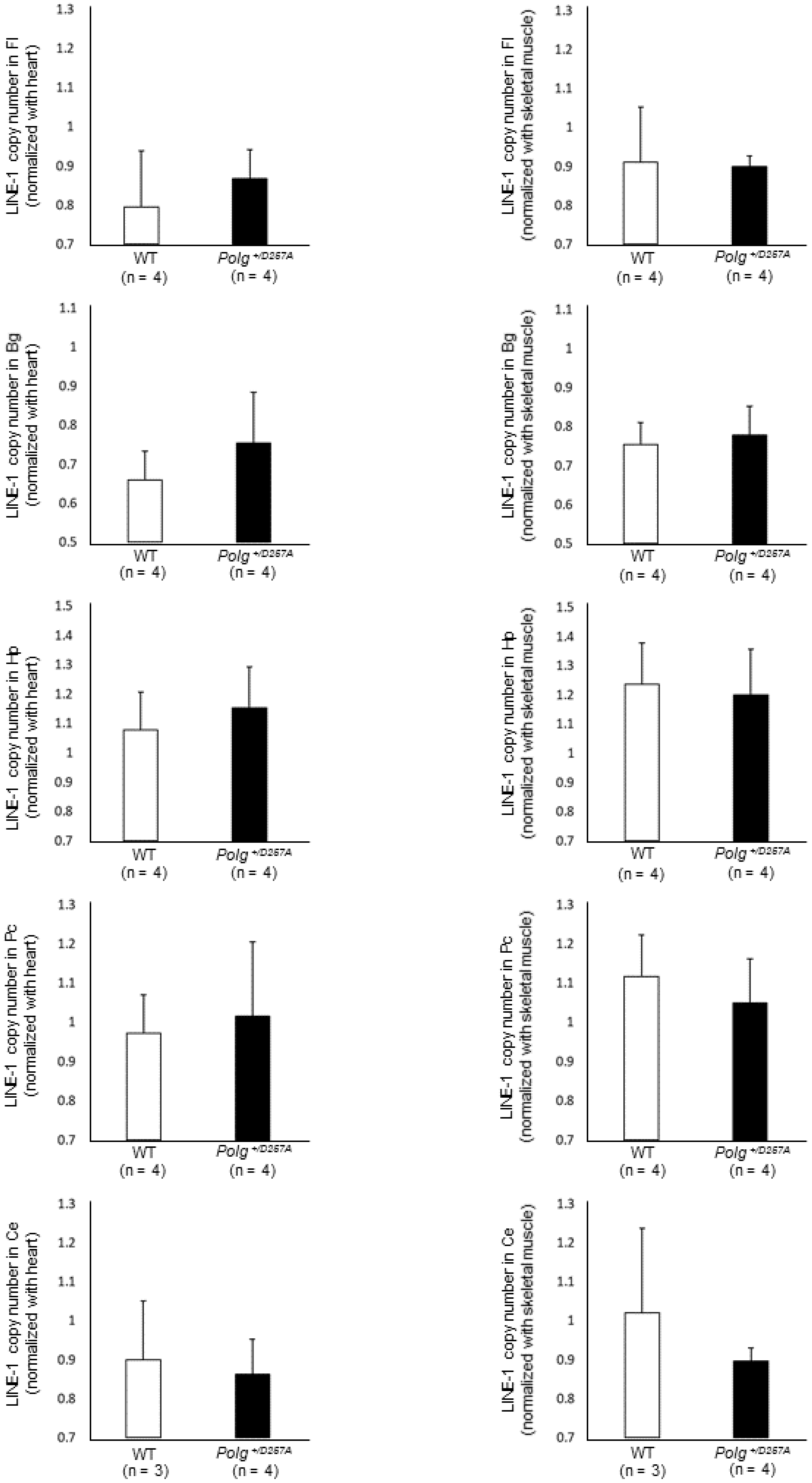

LINE-1 copy number in Fl (normalized with heart)
LINE-1 copy number in Fl (normalized with skeletal muscle)
LINE-1 copy number in Bg (normalized with heart)
LINE-1 copy number in Bg (normalized with skeletal muscle)
LINE-1 copy number in Hp (normalized with heart)
LINE-1 copy number in Hp (normalized with skeletal muscle)
LINE-1 copy number in Pc (normalized with heart)
LINE-1 copy number in Pc (normalized with skeletal muscle)
LINE-1 copy number in Ce (normalized with heart)
LINE-1 copy number in Ce (normalized with skeletal muscle)
WT (n = 4)
WT (n = 3)
Polg +/D257A (n = 4)

(G) GfI_5UTR-ORF1

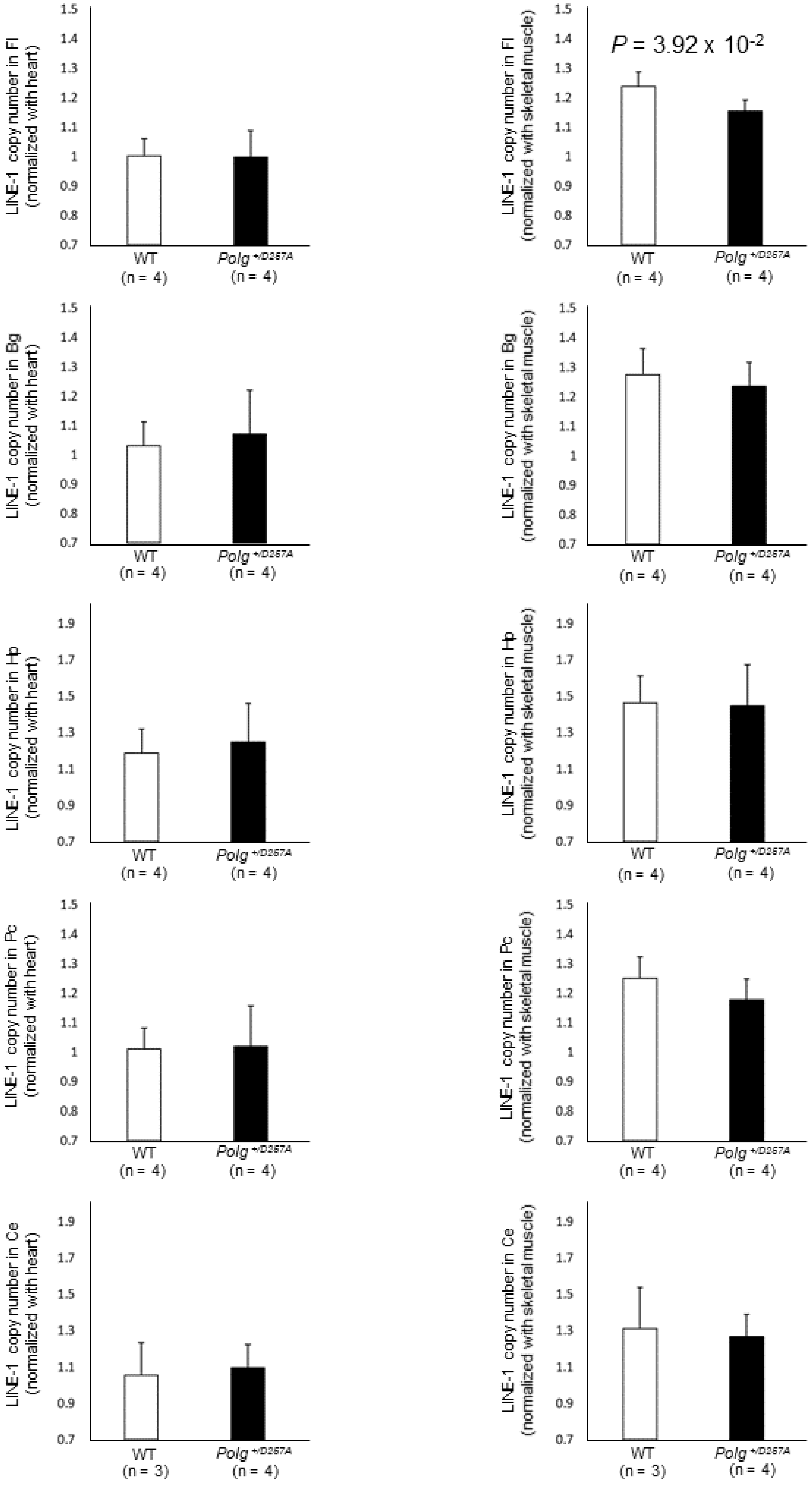

(H) GfII_ORF2

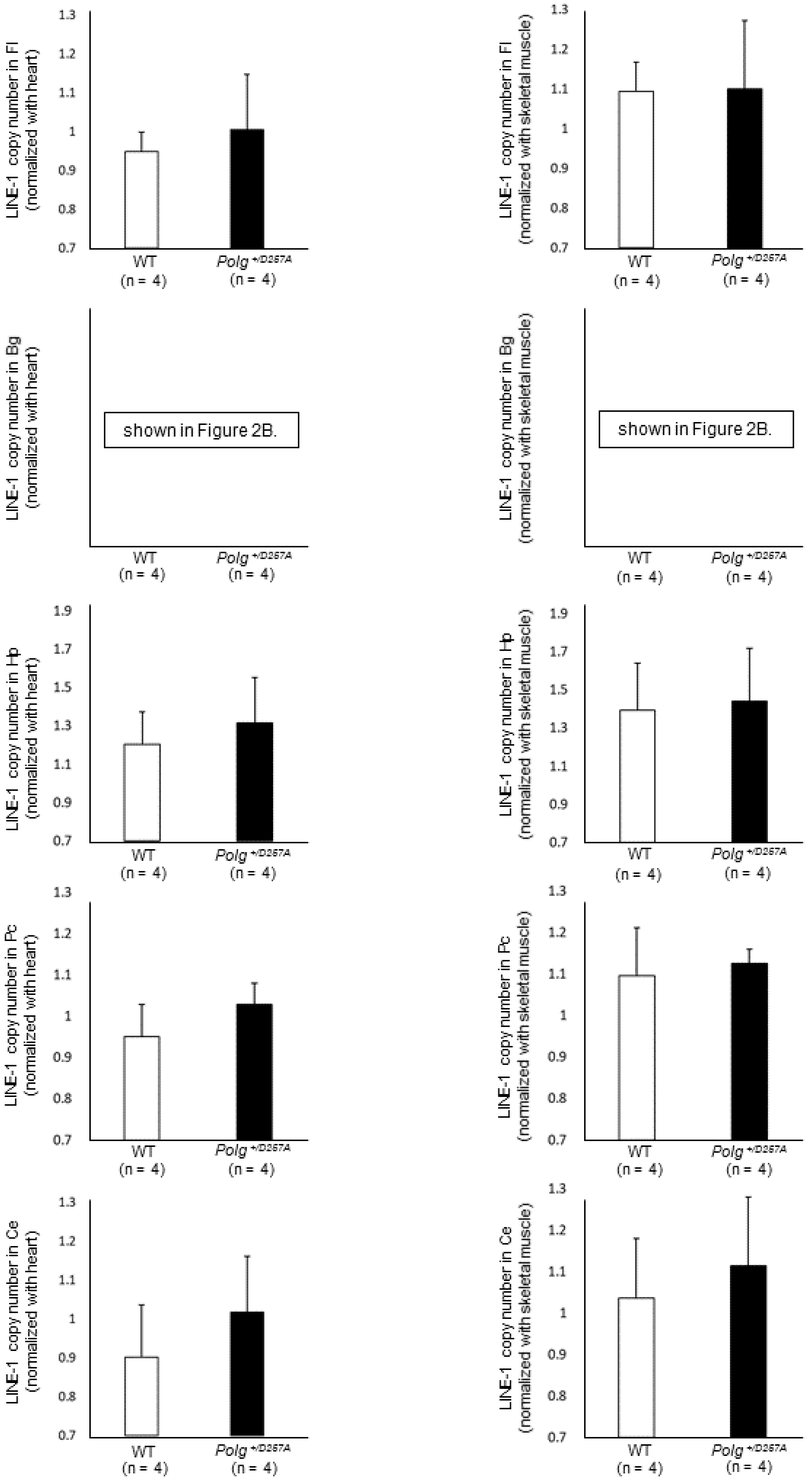

LINE-1 copy number in Fl (normalized with heart)
LINE-1 copy number in Fl (normalized with skeletal muscle)
LINE-1 copy number in Bg (normalized with heart)
shown in Figure 2B.
LINE-1 copy number in Bg (normalized with skeletal muscle)
shown in Figure 2B.
LINE-1 copy number in Hp (normalized with heart)
LINE-1 copy number in Hp (normalized with skeletal muscle)
LINE-1 copy number in Pc (normalized with heart)
LINE-1 copy number in Pc (normalized with skeletal muscle)
LINE-1 copy number in Ce (normalized with heart)
LINE-1 copy number in Ce (normalized with skeletal muscle)
WT (n = 4)
Polg +/D257A (n = 4)

(I) TfII_3UTR

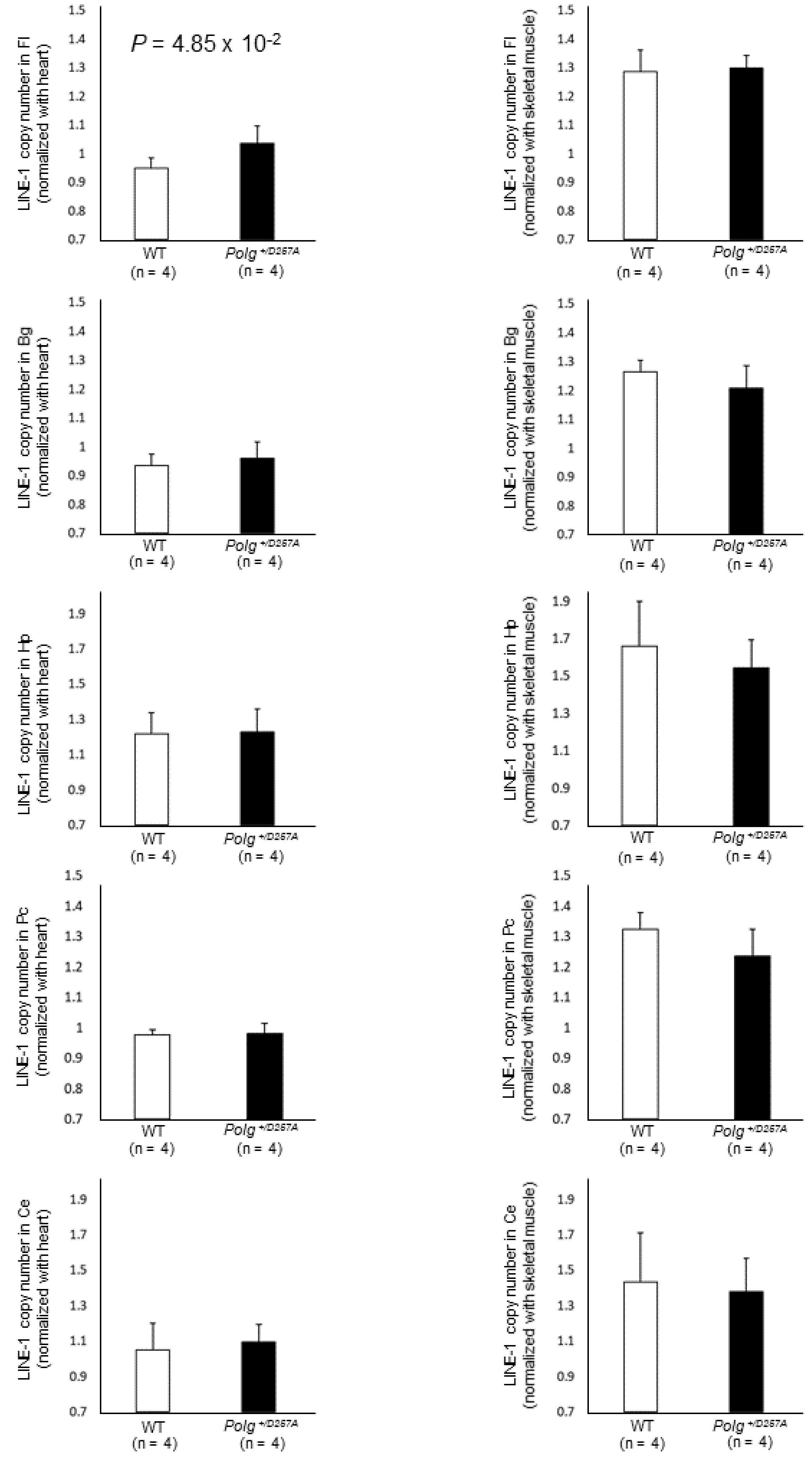

P = 4.85 x 10-2
LINE-1 copy number in Fl (normalized with heart)
LINE-1 copy number in Fl (normalized with skeletal muscle)
LINE-1 copy number in Bg (normalized with heart)
LINE-1 copy number in Bg (normalized with skeletal muscle)
LINE-1 copy number in Hp (normalized with heart)
LINE-1 copy number in Hp (normalized with skeletal muscle)
LINE-1 copy number in Pc (normalized with heart)
LINE-1 copy number in Pc (normalized with skeletal muscle)
LINE-1 copy number in Ce (normalized with heart)
LINE-1 copy number in Ce (normalized with skeletal muscle)
WT (n = 4)
Polg +/D257A (n = 4)
0.7 0.8 0.9 1 1.1 1.2 1.3 1.4 1.5
0.7 0.9 1.1 1.3 1.5 1.7 1.9

Figure S3. LINE-1 copy number detected in 84-week-old *Polg*$^{+/D257A}$ mouse brain. The copy number in the brain was normalized to the heart or skeletal muscle. Data are represented as the mean ± standard deviation. Significant change in Welch's t test is denoted in its p value (others show no significance).

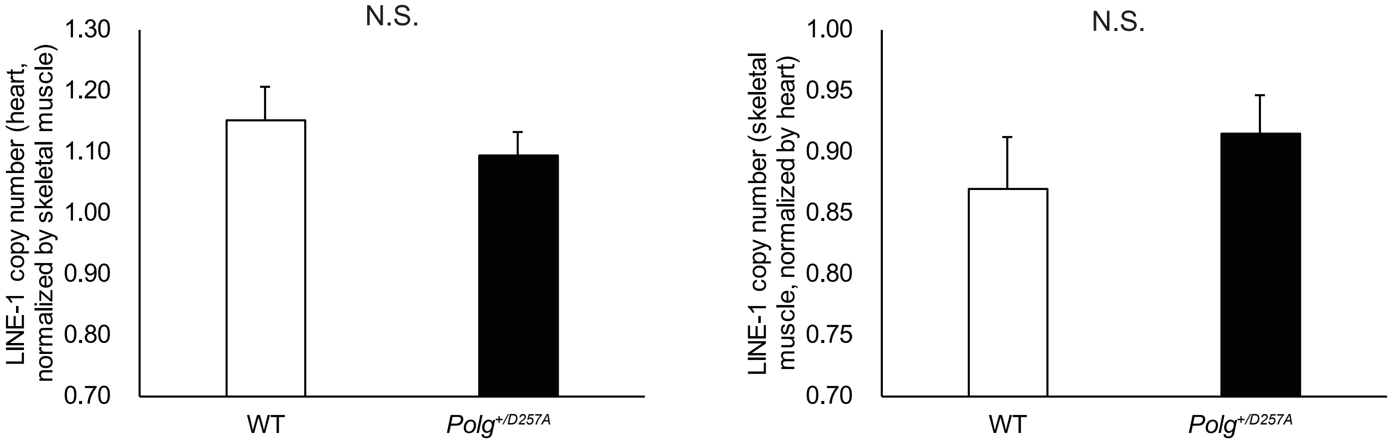

Figure S4. Comparison of LINE-1 contents in heart normalized by skeletal muscle and vice versa. Data of GfII_ORF1 is shown. Data are represented as the mean ± standard deviation. N.S.; not significant in Welch' s t test (P > 0.05), WT; wild-type mice**.**
